# Supplementary figures and images for: A Novel Mastadenovirus from Nyctalus noctula Which Represents a Distinct Evolutionary Branch of Viruses from Bats in Europe
Source: Viruses. 2024 Jul 26;16(8):1207. doi: 10.3390/v16081207 (PMC11359159; doi:10.3390/v16081207)

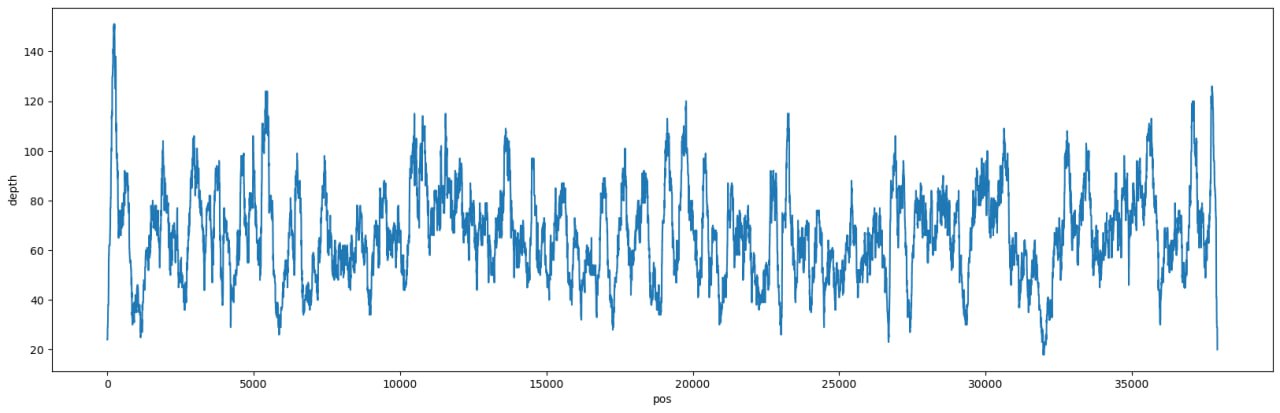

Supplement: Supplementary file 1 [file viruses-16-01207-s001.zip › 26042024_Viruses_Supplementary Figure S1 - Chart for read coverage for assembled genome of Quixote.jpg]

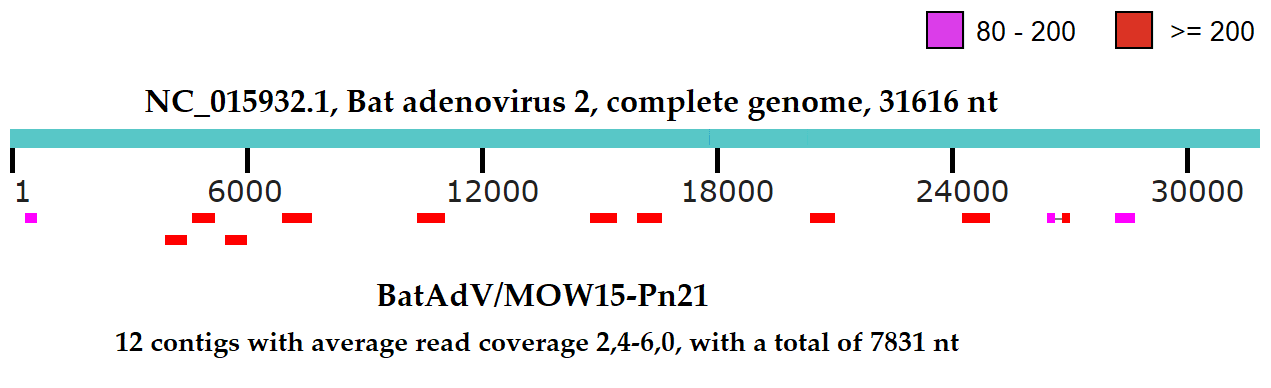

Supplement: Supplementary file 1 [file viruses-16-01207-s001.zip › 26042024_Viruses_Supplementary Figure S2 - MOW15-Pn21 contigs mapping.png]
